# Orchestration of Force Generation and Nuclear Collapse in Apoptotic Cells

**DOI:** 10.3390/ijms221910257

**Published:** 2021-09-23

**Authors:** Bruno Monier, Magali Suzanne

**Affiliations:** Centre de Biologie Intégrative, Unité MCD, CNRS UMR 5077, Université Toulouse III, 118 Route de Narbonne, 31062 Toulouse, France

**Keywords:** apoptosis, cytoskeleton, nucleoskeleton, nuclear envelope blebbing/remodelling/fragmentation, actomyosin

## Abstract

Apoptosis, or programmed cell death, is a form of cell suicide that is extremely important for ridding the body of cells that are no longer required, to protect the body against hazardous cells, such as cancerous ones, and to promote tissue morphogenesis during animal development. Upon reception of a death stimulus, the doomed cell activates biochemical pathways that eventually converge on the activation of dedicated enzymes, caspases. Numerous pieces of information on the biochemical control of the process have been gathered, from the successive events of caspase activation to the identification of their targets, such as lamins, which constitute the nuclear skeleton. Yet, evidence from multiple systems now shows that apoptosis is also a mechanical process, which may even ultimately impinge on the morphogenesis of the surrounding tissues. This mechanical role relies on dramatic actomyosin cytoskeleton remodelling, and on its coupling with the nucleus before nucleus fragmentation. Here, we provide an overview of apoptosis before describing how apoptotic forces could combine with selective caspase-dependent proteolysis to orchestrate nucleus destruction.

## 1. Introduction

**Figure 1:**
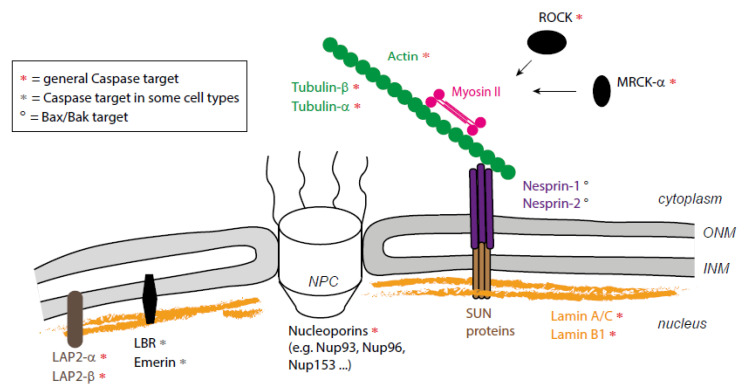
Caspase targets involved in cytoskeleton/nucleus coupling. Schematic representation of the interface between the cytoplasm and the nucleus. Caspases’ targets are indicated by stars. NPC, Nuclear Pore Complex; INM, Inner Nuclear Membrane; ONM, Outer Nuclear Membrane.

The nucleus, which is the biggest and stiffest cell organelle, is kept apart from the cytoplasm by the nuclear envelope. This envelope is made of inner and outer nuclear membranes that join at nuclear pores, which are multiprotein complexes that control nuclear-cytoplasmic trafficking. The nuclear envelope is tightly associated with the nuclear lamina, nucleoskeleton composed essentially of nuclear lamins [1]. Each of the lamin isoforms (classified in type A/C and B) assembles separated meshwork structures as shown recently by cryo-electronic microscopy [2]. This lamina provides structural support to protect the nucleus, maintains nuclear shape and protects the chromatin from external constraints [3,4,5]. The lamina is connected to the cytoskeleton through a macromolecular complex, known as the LINC (Linker of nucleoskeleton and cytoskeleton) complex, that spans the nuclear envelope [6]. The LINC is a bipartite complex composed of nesprin and SUN proteins, respectively, embedded in the external and internal nuclear envelope, and interacting in the space between those two membranes. SUN proteins also interact with lamins in the nucleus, while nesprins interact directly or indirectly with F-actin, microtubules or intermediate filaments in the cytosol [6] (Figure 1). Importantly, nuclear fragmentation is one of the classical hallmarks of apoptosis (or programmed cell death) originally described by Kerr et al. [7], although the mechanisms underlying this process have long remained unclear. Here, we briefly introduce apoptosis before gathering data spanning almost three decades in order to propose a conceptual framework for nucleus dismantling by the coordinated action of the proteolytic activity of caspase enzymes and cytoskeleton-dependent forces. We point out questions of interest for future research, ranging from the identification of the molecular actors mediating the interaction between the cytoskeleton and the nucleus in dying cells to the possible consequences of apoptotic nuclear mechanotransduction. Finally, we draw attention to the fact that the nucleus is also necessary in dying cells to generate forces that shape tissues during development.

## 2. Apoptosis: A Stepwise Dynamic Process

Apoptosis, also known as programmed cell death, is the cellular process by which cells die in a coordinated fashion, supposedly with limited immunological impact, if any [8]. Apoptosis is orchestrated by caspases, cysteine proteases that are highly conserved across evolution [9]. Intrinsic or extrinsic death signals, e.g., TNF-alpha, converge on activation of upstream caspases that, in turn, cleave and activate executioner caspases, such as caspase-3, 6 and 7. This cascade of caspase activation leads to the coordinated cleavage of (at least) a few hundred of the protein targets [10] that will orchestrate the stepwise destruction of the cell (some of them are presented in Figure 1, indicated by stars).

This type of death leads to dramatic cellular remodelling, including cellular shrinkage, blebbing and fragmentation into apoptotic bodies, small corpses that are cleared by phagocytes or living neighbours [7]. Cell shape changes are mediated by remodelling of the cytoskeleton and the generation of mechanical forces. Not surprisingly, the dramatic apoptotic cell shape changes are associated with altered mechanics, and many cytoskeletal proteins are targets of caspases [11]. For instance, caspase-mediated cleavage of the kinase ROCK irreversibly relieves its auto-inhibition. Activated ROCK then mediates phosphorylation of myosin II Regulatory Light Chain (MRLC), causing a hyperactivation of non-muscle myosin II, from which originates the dynamic blebbing of the dying cell membrane, and eventually the formation of the apoptotic bodies [12,13,14,15]. Interestingly, the inhibition of apoptotic cell contractility through the expression of a non-cleavable form of ROCK has recently been shown to delay the elimination of cell debris, which leads to sterile inflammation and has been associated with tumour suppression [16].

These observations were made essentially in cultured cells where apoptotic cells detach from their substrate before fragmenting. However, when apoptosis occurs in epithelial cells, the dying cell keeps strong adhesion with its neighbours [17] and is progressively expulsed from the epithelial sheet through a mechanical process called cell extrusion [18]. Cell extrusion is concomitant with the execution of apoptosis and involves an active contribution of both the dying cell and its living neighbours. First actomyosin forms an internal ring in the extruding cell leading to apical constriction. The generation of an apical actin ring in extruding cells is a consequence of the cleavage of the kinase MRCK-alpha by caspases [19]. Then, their neighbours form a multicellular ring of actomyosin around the extruding cell, preventing the formation of any gap and thus ensuring the maintenance of the barrier function of the epithelium while the dying cell is expulsed [18]. The neighbours also form lamellipodia protrusions, which participate in the basal sealing of the epithelium [20]. Hence, the dismantlement of doomed cells is a combination of tightly linked biochemical and mechanical events.

## 3. A combination of Proteolytic and Mechanical Events Leads to Nucleus Destruction

In parallel to these cellular rearrangements, the nucleus is highly remodelled during apoptosis. Apoptotic nucleus remodelling includes increased nuclear permeability, chromatin condensation, DNA fragmentation, nuclear pore clustering, nuclear envelope blebbing and eventually fragmentation [21]. This orchestrated demolition relies on the targeting of different nuclear components by the caspases: (1) the nucleopore components, which results in increased nuclear permeability; (2) the nuclease inhibitor ICAD, which results in the activation of CAD/DFF40 and the degradation of the DNA; but also (3) structural components such as lamins [21,22].

### 3.1. Nuclear Blebbing, an Early Step of Apoptotic Nucleus Dynamics

An early event of apoptosis, described recently in mouse embryonic fibroblasts, is the formation of nuclear bubbles at the nuclear periphery. These bubbles appear in regions depleted of lamins. They contain nuclear proteins, rupture and discharge their content in the cytosol [21,23]. This is reminiscent of what has been observed in mammalian cells when migrating in a confined environment [24,25] or in pathological contexts such as cancer cells or laminopathy [26]. In these cells, a nuclear envelope ruptures locally, a process that is favoured by lamin reduction and mechanical compression [24,25,26]. Nuclear envelope ruptures coincide with chromatin protrusion, DNA damage and nuclear fragmentation [24,25]. These ruptures are associated with the assembly of contractile actin bundles and depend on the contractile activity of actomyosin and the integrity of the LINC complex [25,26]. In these cells, the ruptures are only transient and are repaired by the ESCRT (Endosomal Sorting Complexes Required for Transport) machinery [24,25]. These studies point to the importance of nucleus mechanical response to ensure the protection of the genome [4]. During migration, to circumvent nuclear deformation and potential DNA damage, the nuclear envelope must possess the right balance between stiffness and plasticity to navigate through dense regions [27]. A-type lamins play an important role in this balance since a nucleus can only be deformed efficiently if its level of A-type lamin is sufficiently low. However, if the level of lamin A is too low, this can lead to migration-associated apoptosis [28], showing that mechanical constraints, when too high to be sustained by a given nucleus, will lead to irreversible damage and subsequent cell death.

Could this DNA damage sensitivity to high-level forces be exploited by cells committed to programmed cell death? One may hypothesise that increasing tension through caspase-dependent myosin activation and connection of the cytoskeleton to the nuclear envelope will speed up DNA fragmentation, eventually facilitating DNA fragmentation. The observation of these blebs in apoptotic cells and in constrained migrating cells suggests that the formation of nuclear bubbles could be a general mechanism induced in response to stress. However, nuclear ruptures have also been observed in differentiating cells, such as during mouse erythropoiesis [29]. In this context, nuclear opening is mediated by caspases and constitutes an essential step for the enucleation. Interestingly, nuclear opening in erythroblasts also coincides with chromatin condensation. Thus, local weakening of the nuclear lamina and nuclear opening occur not only in stress conditions but could also contribute to the regulation of chromatin condensation during differentiation.

### 3.2. Nuclear Dismantling

Following this first step of local depletion of lamins, the apoptotic nucleus becomes totally dismantled and fragments. Early studies proposed that caspase-mediated cleavage of nuclear structural proteins was sufficient for apoptotic nuclear disintegration [30]. Yet, subsequent work demonstrated that if the expression of non-cleavable forms of A- and/or B-type lamins delays apoptosis, it does not always block nuclear dismantling [31]. However, one should keep in mind that lamins network is composed of separated meshwork structures, and non-cleavable lamins were expressed in the presence of endogenous lamins that could still be processed by caspases. This may be sufficient to soften the lamina and finally lead to nucleus fragmentation, although with a delay. This suggests that lamin breakdown by proteolysis could facilitate nuclear breakdown but that additional mechanisms might be at work. Interestingly, in the course of their analysis of apoptotic cell blebbing, Coleman and colleagues observed that ROCK inhibition also prevented the eventual relocalisation of fragmented DNA into apoptotic bodies [12]. This led Croft and colleagues to report later on that nuclear fragmentation necessitates actomyosin cytoskeleton contractility on top of lamin cleavage [32]. Indeed, they reported that ROCK inhibition or F-actin destabilization, which block TNF-alpha induced apoptotic blebbing, also abolish nucleus fragmentation. Those drugs do not alter caspase activation, indicating that they do not interfere with apoptosis induction. A similar phenotype was obtained by abolishing myosin II ATPase activity using Blebbistatin or by expressing a non-phosphorylable form of the regulatory light chain, MRLC. Together, those results indicate that an intact and contractile actomyosin cytoskeleton is necessary to mediate nucleus fragmentation.

Because caspase-dependent lamin cleavage is important for nucleus dismantling [21,31], Croft and colleagues investigated whether ROCK inhibition affects lamin cleavage [32]. It turned out not to be the case, and cleavage of additional key nuclear molecules such as the nucleopore component Nup153 or the lamin-associated protein LAP2-alpha, is also unaffected by ROCK inhibition, while caspase inhibition totally abrogated lamin cleavage. Then, they tested whether actomyosin hypercontractility could be sufficient to promote nucleus disintegration in non-apoptotic cells. Experimental ROCK activation proved sufficient to change the shape of nuclei, rendering them occasionally smaller and distorted, but this condition did not lead to nucleus fragmentation. However, in laminA/C depleted cells, which have a weakened nucleoskeleton (and in which caspases’ activity is blocked), forced ROCK activation led to nuclei disruption. Altogether, those important experiments demonstrate that nuclear dismantling during apoptosis is the result of two complementary actions: proteolytic weakening of the lamina and ROCK-induced actomyosin contraction, both being controlled by caspase activity.

## 4. Actomyosin-Nucleus Coupling before Fragmentation

While the work of Croft et al. demonstrates the importance of actomyosin contractility in nucleus fragmentation [32], how the cytoskeleton reorganises the need to fragilise the apoptotic nucleus remains unknown. It was proposed that a cytoplasmic meshwork of actin filaments surrounds the nucleus, whose contraction could tear it off, but support for such a hypothesis is lacking. A possible alternative mechanism emerged recently from a study identifying the cellular mechanism underlying apoptotic force generation during *Drosophila* leg development [33].

**Figure 2:**
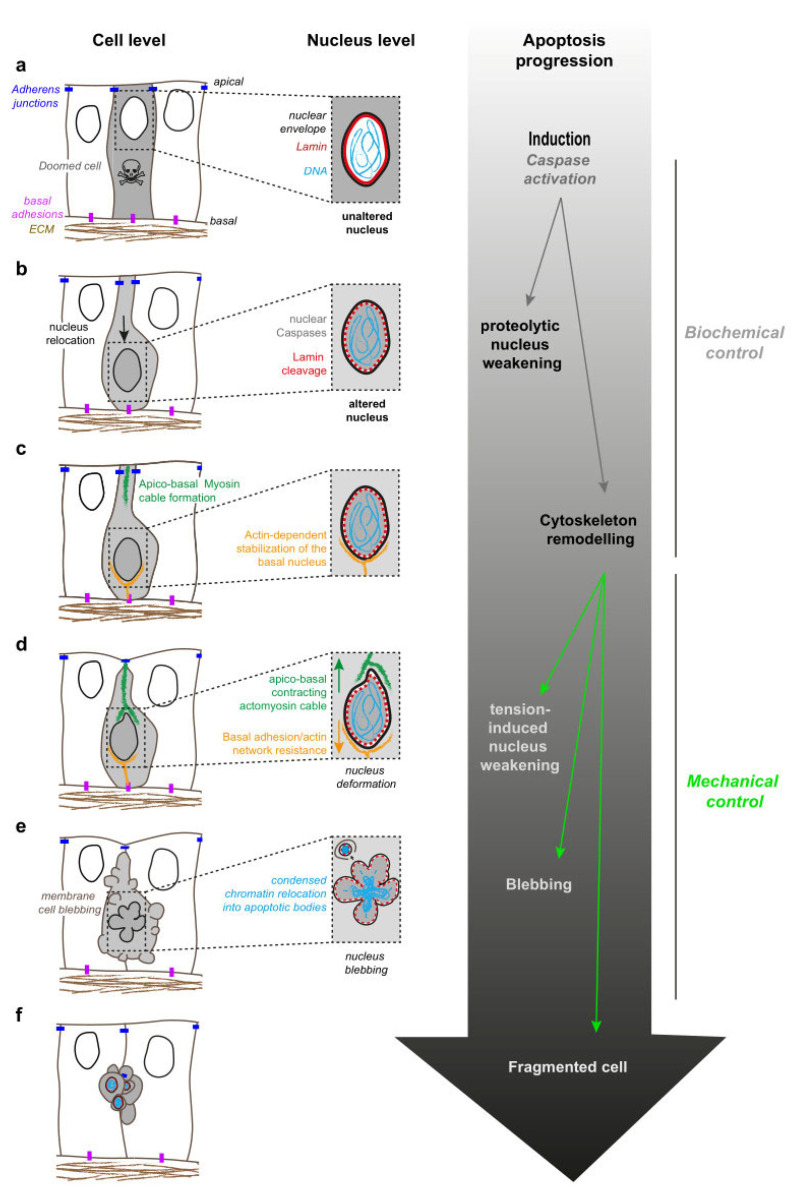
A model of biochemical and mechanical cooperation during apoptotic nucleus dismantling. Schematic representation of cell and nucleus dynamics (left and middle columns, respectively) gained from cells undergoing apoptosis in culture and in vivo. Contractile actomyosin structures are depicted in green, while non-contracting actin networks are shown in orange. Main steps driving nucleus dismantling are indicated on the right. Note that biochemical and mechanical aspects of apoptosis are represented subsequently for clarity, although proteolysis is not restricted to early stages of apoptosis. The main apoptotic stages are divided into caspase activation (**a**), nuclear relocation and apical constriction (**b**), nucleus basal anchoring and myosin II cable apical growth (**c**), myosin II cable/nucleus coupling, apico-basal force generation and nucleus deformation (**d**), cell and nucleus blebbing (**e**) and finally cell and nucleus fragmentation (**f**).

**Figure 3:**
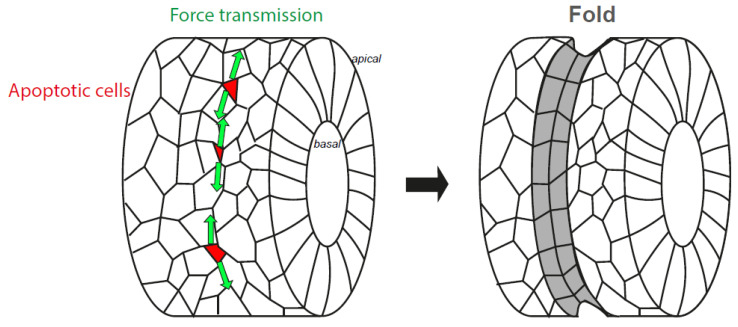
Impact of apoptosis on epithelium folding. Schematic representations of the *Drosophila* leg disc before (left) and after (right) fold formation. This tissue initially forms a cylinder and apoptotic cells are represented in red. The apoptotic forces are then produced, transmitted to the neighbouring tissue (green arrows), leading to the formation of a fold (grey).

During fly development, some cells of the developing leg, or leg imaginal disc (an epithelial cylindrical monolayer that ultimately gives rise to the adult appendage), die through apoptosis along the circumferential axis, a process necessary for the formation of the adult leg joints [34]. At the early stages of the execution phase, apoptotic cells maintain their apical adherences with their neighbours, constrict their apex and subsequently form a transient apico-basal actomyosin structure, hereafter referred to a “myosin cable” (see Figure 2c,d, cell level). When this cable contracts, it generates a force in these dying cells. This traction force is sensed by living neighbours, which react by accumulating apical myosin II, constricting their apex and eventually forming a fold that prefigures the adult joint [35] (see Figure 3).

Surprisingly, the apoptotic myosin cable runs from the apical domain to the middle of the cell, raising the question of its connection to additional cellular components [33,35]. Analysis of the shape of the leg disc cells revealed that while living cells possess a large apical cell body with a thin basal cell extension connecting to the underlying extracellular matrix, dying cells have the reverse morphology. This turned out to be the consequence of nucleus localization within the tissue: in this pseudostratified epithelium, nuclei accumulate in the apical half of the epithelium, with the exception of the nuclei of apoptotic cells that relocalise on the basal half of the epithelium. There, the nucleus is stabilised by a F-actin network connected to basal adhesions. At this early apoptotic stage, chromatin organization appears unaltered (as assayed by DAPI staining), but lamin levels slightly drop, consistently with the presence of active executioner caspase Dcp1 within the nucleus (Figure 2b,c) [33].

Interestingly, the basal side of the myosin cable lies near to the relocated nucleus, suggesting an interaction between them. Such an interaction was demonstrated through laser ablation experiments: cutting the apico-basal myosin cable leads, on one side, to the apical retraction of the surface of the epithelium and, on the other side, to the basal retraction of the nucleus. Moreover, when the myosin cable starts contracting, the apical surface of the apoptotic nucleus deforms while it moves back towards the apical side before eventually becoming fragmented (Figure 2d) [33]. A direct impact of the force on nucleus fragmentation has not been tested in this study but, given the results reported previously [32], we propose that caspase-mediated lamin cleavage (likely leading to the decrease in lamin levels observed) would cooperate with the apico-basal force applied onto the nucleus to tear it off. Such a force would be the result, on one side, of a contractile cable linked to the apical surface, likely at adherens junctions, and on the opposite side of a basal resisting nucleus anchored to a F-actin network linked to basal adhesions (Figure 2d). This model could be tested by capturing the dynamics of apoptotic nucleus fragmentation in living tissues in various conditions: following the expression of non-cleavable lamins or by abrogating the generation of the apoptotic force, which could be achieved by blocking nucleus positioning or weakening basal adhesions as previously reported [33].

Of note, the apico-basal myosin cable that connects to the nucleus is described here in a context where apoptosis plays a morphogenetic role, i.e., triggering leg fold formation (see below). However, a contractile actin cable connecting the apoptotic nucleus while it undergoes a similar basal-to-apical movement before fragmentation was also reported during non-morphogenetic, UV-induced cell death in the *Drosophila* wing disc [36]. This suggests that, at least in *Drosophila*, the contraction of a force-generating actomyosin apico-basal cable connected to the nucleus before its fragmentation could be a general feature of apoptosis.

## 5. Could Apoptotic Microtubules Also Contribute to Nuclear Fragmentation?

While the actomyosin cytoskeleton has gained some attention during the apoptotic process, far less is known about the role of microtubules during cell death, especially regarding their interaction with the dismantling nucleus. It comes in part from observations that some microtubules’ components are caspases’ targets, and that the microtubule cytoskeleton is dismantled relatively early during the execution phase (likely at the time of cell detachment from the substrate, before fragmentation) [37]. However, microtubules may play a role before being dismantled. Furthermore, a late network of microtubules is eventually rebuilt in some cell types, although it might not rely upon centrosomes since gamma-tubulin is absent. It is observed in fragmenting cells, participates in chromatin fragments dispersal [37] and also temporarily protects key molecular targets of caspases, such as E-Cadherin or Integrin subunits, from precocious cleavage and consequently uncontrolled necrosis [38]. The building of the “late” apoptotic microtubules network is under the control of the small GTPase Ran [39,40]. Active RanGTP is released form the nucleus to the apoptotic cytoplasm where it triggers microtubule nucleation, together with the spindle-assembling factor TXP2. RanGTP relocation, and therefore subsequent microtubule network rebuilding, are dependent upon actomyosin activity, since they are blocked by blebbistatin or Y27632 treatment.

Interestingly, microtubules are also under the control of Ran and TXP2 during mitosis [41], another process during which the nuclear envelope is usually transiently dismantled. Moreover, microtubules associate in tight connection with the nuclear envelope during mitosis. In this case, Dynein-dependent pulling forces applied on astral microtubules lead to the nuclear envelope invagination around centrosomes, facilitating the subsequent nuclear envelope fenestration. Then, microtubule-dependent pulling on LINC complexes participate in the remodelling of the nuclear envelope during mitosis [27]. This observation led to consider a potential link between microtubules-dependent forces and the dismantling of the apoptotic nuclear envelope. A recent report, conducted on living cancerous cells in culture, suggests that this might be the case. Indeed, in several ovarian cancer cells, drug-dependent stabilization of microtubules, especially using paclitaxel, causes a “multi-micronucleation” phenotype [42], with stabilised microtubules associating with fragments of the nucleus. The same treatment, however, does not affect the shape of the nucleus in non-cancerous cells. It was shown previously that the nuclear envelope of ovarian cancer cells becomes deformable due to the loss of lamin A/C [43,44]. Consistently, the authors reported that *lamin A* ovarian mutant cells become sensitive to paclitaxel treatment, while overexpression of lamin A in ovarian cancer cells protected against paclitaxel-induced nuclear fragmentation. Those results regarding nucleus/microtubules interaction, although obtained in living cells, are strikingly reminiscent of those of Croft and colleagues [32], in which caspase-dependent ROCK activation promotes the nuclear envelope fragmentation following lamin network weakening. Microtubules were not required in the cell type used in this particular study, and possibly a role for microtubules might be cell-type specific. Yet, we believe microtubules should be given far more interest in the future regarding their possible impact on nuclear mechanics during the apoptotic process.

## 6. What Mechanism for Nucleus/Cytoskeleton Interaction during Cell Death?

One critical question regarding the apoptotic nucleus is to understand how it is anchored to the cytoskeleton. Is the LINC complex connecting the cytoskeleton to the nucleus during cell death? In the fly, two nesprins (external components of the LINC complex) exist, Klarsicht and Msp300. Klarsicht, the nesprin that associates with microtubules, is perinuclear and is necessary for basal relocalisation of the apoptotic nucleus [33], suggesting a role of the early microtubule network in nuclear positioning in apoptotic cells. Following basal relocation, the apoptotic nucleus interacts with the actomyosin cable [33], an interaction that should be mediated by Msp-300, the fly actin-binding nesprin. Surprisingly, Msp-300 localisation was not reported to be perinuclear in the fly leg, raising the question of its implication in apoptotic myosin cable/nucleus anchoring. Moreover, in mammalian cells, recent evidence shows that the perinuclear localisation of several nesprins is lost following apoptotic stimulus [45], although the exact timing of nesprin relocation with respect to apoptosis execution and nucleus fragmentation remains to be characterised. Identifying the molecules connecting the cytoskeleton to the nuclear envelope and characterising their dynamics during apoptosis is therefore a key task for the future.

Interestingly, during mitosis, a direct link between cytoskeletal and intranuclear components is observed, even in cells that undergo closed mitosis, where the nuclear envelope is maintained. Indeed, in closed mitosis, the nuclear envelope becomes permeable to give access to tubulin and microtubule-nucleating centres to the intranuclear region and construct the mitotic spindle [46]. Could something similar take place during apoptosis? In this process, the nuclear membrane becomes permeable following nucleoskeleton weakening and nucleopore alteration [21,47]. Altered nucleopores or local nuclear membrane ruptures could create entry points for the actomyosin cable formed in epithelial cells. As a result, the actomyosin cable would enter the nucleus and bind to nuclear constituents such as lamins, which have been reported to interact directly with actin [48]. This speculative model obviously needs support from in vivo studies.

## 7. A Necessity to Understand Nuclear Mechanotransduction during Cell Death

Various types of forces may impact the nucleus during apoptosis. The first events in dying epithelial cells are apical constriction and basal nucleus relocation [33,35]. This is reminiscent of what has been described in cells of the *Drosophila* mesoderm. Indeed, these cells, although non-dying, will undergo invagination and initiate their change in shape by apical constriction [49]. Apical constriction of mesodermal cells was demonstrated to create an apico-basal flux of cytoplasm [50] that pushes the nucleus basally [51]. Cytoplasmic flux is not sufficient in *Drosophila* dying cells to relocate the nucleus, since it remains apical in the absence of the LINC (suggestive of an active apical-to-basal transport). Yet, apical constriction-induced flow of cytoplasm could nevertheless transiently apply compressive forces onto the apical side of the nucleus at early apoptotic stages. Next, as described above, the basally relocated apoptotic nucleus becomes coupled to the actomyosin cytoskeleton. The apoptotic nucleus moves back apically while myosin II contracts, transiently deforming the apical nuclear envelope. Finally, after experiencing tensile forces and stretching, the dying nucleus may once again experience compressive forces while the dying cell shrinks at late apoptotic stages.

In living cells, it is now widely accepted that nuclei have the capacity to respond to forces or mechanical constraints and modify their transcriptional activity in response to extracellular matrix stiffness, shear stress or cell contractility [4,52]. Forces can impact the nucleus at different levels, modifying either nuclear envelope permeability by altering nucleopores’ shapes [53], nuclear envelope stiffness (measured with magnetic beads or by AFM, see [54,55]) or chromatin compaction [56,57,58,59,60]. The lamina, which maintains the shape of the nucleus, also plays a key role in nuclear mechanotransduction. Lamins, tightly connected to the cytoskeleton through the LINC complex [61] are also associated with peripheral heterochromatin at the nuclear envelope [1], either directly or through association with proteins from the inner nuclear membrane such as LAP1, LAP2, LBR, Emerin or Man1. Interestingly, Emerin has been shown to mediate nuclear mechanical response to tension in isolated nuclei, while the depletion of LAP2 or MAN1 does not affect nuclear stiffening [54]. Due to this tight association of the lamina with the chromatin, the nuclear lamina, on top of its protective function, could also transmit forces and have an impact on chromatin organisation and compaction [62].

This raises the question whether forces can trigger nuclear mechanotransduction responses in apoptotic cells before promoting nucleus fragmentation. In *Drosophila* dying cells, DAPI staining suggests that chromatin organization is not altered yet, or only weakly altered, at the time that the force starts to be applied onto the nucleus. Could the force quickly stiffen the nuclear envelope, as previously reported [54], and indirectly impact on global transcription shut down or chromatin condensation? Of note, in mammalian cultured cells, chromatin still condenses in the absence of forces [32], which may appear in contradiction with a role of force in chromatin condensation at first glance. However, it may be more complex and dynamic, and forces may only enhance the kinetics of chromatin remodelling, a parameter that could not be captured with studies on fixed dying cells. The analysis of apoptotic nucleus dynamics in living tissues, thanks to recently developed caspase sensors [36,63], could provide a means to address such questions.

## 8. The Nucleus, from an Organelle under Force to an Organelle Necessary to Generate Morphogenetic Forces

If applying forces to the nucleus may help to shut down transcription, as mentioned above, it may have another role. In non-dying cells, the state of chromatin was shown to modulate nuclear mechanical properties: nuclear deformability is, respectively, favoured or decreased by chromatin decondensation or condensation [59,64,65,66,67,68]. In epithelial dying cells, we speculate that the nucleus could transiently become less deformable before its eventual fragmentation. Thereby, it could provide more resistance to the actomyosin cable anchored to it, creating a positive feedback loop to enhance the efficiency of the apico-basal myosin cable contractility. Obviously, those forces could reach a threshold necessary to dismantle the nucleus, but it could also have collateral effects. Indeed, forces generated inside the dying cells, thanks to nucleus-cytoskeleton coupling, are also transmitted to the neighbouring cells via the not-yet dismantled cell–cell junctions [17,35]. These forces then propagate at the level of adherens junctions in the neighbouring tissue, triggering apical cell shape changes and myosin accumulation at a distance from the dying cell. In consequence, the multiplication of local apoptotic events during *Drosophila* leg formation causes tissue remodelling and fold formation [35]. Impairing either nucleus relocation or basal anchorage in these cells greatly diminishes force generation at the cell level, which causes the loss or malformation of some leg folds. Thus, apoptotic forces dependent on the nucleus–cytoskeleton interaction are morphogenetic [33].

Several recent works demonstrate that tissue-scale patterns and coupling of the actomyosin cytoskeleton provide robustness during tissue folding [69,70,71]. In particular, during leg folding, myosin II is planar polarised at adherens junctions, orienting force propagation along the fold axis. This bias in force propagation overcomes the impact of mechanical noise due to non-related morphogenesis events taking place in the vicinity of the fold domain. Indeed, abolishing myosin polarity (without affecting the pattern of apoptosis or the ability of dying cells to generate forces) often leads to deviating folds. Since junctional myosin accumulation over time is dependent on apoptosis [35], one may wonder about the possible dependence of myosin polarity upon apoptotic forces. If so, dying cells could not only act as a trigger to initiate folding though the release of mechanical forces, but also stimulate myosin polarity at the tissue level to provide robustness to morphogenesis. Both effects would rely on the efficient release of apoptotic forces, which are strictly dependent on cytoskeleton–nucleus coupling in dying cells [33]. Of note, the importance of the nucleus in tissue mechanics is not limited to leg fold formation. Indeed, a recent study on the role of the LINC demonstrated that its knockdown in glandular acini leads to altered morphogenesis, since the lumen collapses in the absence of the LINC. However, in that case, this is due to an upregulation of ROCK/myosin-dependent tension [72]. Hence, the nucleus, in addition to being a target of intracellular forces, is a key actor in morphogenetic events that deserves in-depth characterisation in order to identify general vs. cell-type-specific roles.

## 9. Conclusions

Altogether, the data we gathered here highlight the complementarity of biochemical and mechanical signals in the orchestrated destruction of the apoptotic nucleus (Figure 2). Multiple questions remain unaddressed, especially regarding the coupling of the cytoskeleton to the nucleus during cell death, and the exact contribution of microtubules. Finally, if the nucleus is submitted to forces in a large variety of contexts beyond apoptosis, such as migration or mitosis, recent data also reveal an unexpected mechanical function of the nucleus in force generation that needs further investigation.
